# Parental Mosaicism in *PAX6* Causes Intra-Familial Variability: Implications for Genetic Counseling of Congenital Aniridia and Microphthalmia

**DOI:** 10.3389/fgene.2018.00479

**Published:** 2018-10-17

**Authors:** María Tarilonte, Matías Morín, Patricia Ramos, Marta Galdós, Fiona Blanco-Kelly, Cristina Villaverde, Dolores Rey-Zamora, Gema Rebolleda, Francisco J. Muñoz-Negrete, Saoud Tahsin-Swafiri, Blanca Gener, Miguel-Angel Moreno-Pelayo, Carmen Ayuso, Manuela Villamar, Marta Corton

**Affiliations:** ^1^Department of Genetics and Genomics, Instituto de Investigación Sanitaria de la Fundación Jiménez Díaz, University Hospital – Universidad Autónoma de Madrid, Madrid, Spain; ^2^Servicio de Genética, Instituto Ramón y Cajal de Investigación Sanitaria, Hospital Universitario Ramón y Cajal, Madrid, Spain; ^3^Centre for Biomedical Network Research on Rare Diseases, Instituto de Salud Carlos III, Madrid, Spain; ^4^Department of Ophthalmology, Cruces University Hospital, Bilbao, Spain; ^5^Department of Glaucoma, Instituto Ramón y Cajal de Investigación Sanitaria, Hospital Universitario Ramón y Cajal, Madrid, Spain; ^6^Department of Genetics, BioCruces Health Research Institute, Cruces University Hospital, Bilbao, Spain

**Keywords:** parental mosaicism, *PAX6*, aniridia, variable expressivity, microphthalmia, post-zygotic variants

## Abstract

Mutations in *PAX6* are involved in several developmental eye disorders. These disorders have considerable phenotypic variability, ranging from panocular forms of congenital aniridia and microphthalmia to isolated anomalies of the anterior or posterior segment. Here, we describe 3 families with variable inter-generational ocular expression of aniridia, iris coloboma, or microphthalmia, and an unusual transmission of *PAX6* mutations from an unaffected or mildly affected parent; all of which raised suspicion of gonosomal mosaicism. We first identified two previously known nonsense mutations and one novel likely pathogenic missense variant in *PAX6* in probands by means of targeted NGS. The subsequent segregation analysis by Sanger sequencing evidenced the presence of highly probable mosaic events in paternal blood samples. Mosaicism was further confirmed by droplet digital PCR analysis in several somatic tissues of mosaic fathers. Quantification of the mutant allele fraction in parental samples showed a marked deviation from 50%, with a range between 12 and 29% depending on cell type. Gonosomal mosaicsm was definitively confirmed in one of the families thanks to the availability of a sperm sample from the mosaic father. Thus, the recurrence risk in this family was estimated to be about one-third. This is the first report confirming parental *PAX6* mosaicism as a cause of disease recurrence in aniridia and other related phenotypes. In addition, we demonstrated that post-zygotic mosaicism is a frequent and underestimated pathogenic mechanism in aniridia, explaining intra-familial phenotypic variability in many cases. Our findings may have substantial implications for genetic counseling in congenital aniridia. Thus, we also highlight the importance of comprehensive genetic screening of parents for new sporadic cases with aniridia or related developmental eye disease to more accurately assess recurrence risk. In conclusion, somatic and/or gonosomal mosaicism should be taken into consideration as a genetic factor to explain not only families with unaffected parents despite multiple affected children but also variable expressivity, apparent *de novo* cases, and even uncharacterized cases of aniridia and related developmental eye disorders, apparently lacking *PAX6* mutations.

## Introduction

*PAX6* encodes a highly conserved homeodomain-containing transcription factor that plays pivotal roles in normal ocular and neural development (Cvekl and Callaerts, 2017). Dominant *PAX6* mutations lead to a spectrum of ocular developmental anomalies (ODAs) depending on the mutation type and gene dosage (van Heyningen and Williamson, 2002). *PAX6* haploinsufficiency, which results from loss-of-function variants or 11p13 microdeletions involving this gene or their 5′ regulatory regions, is the major cause of congenital aniridia (MIM# 106210) (Hingorani et al., 2012). By contrast, missense mutations usually exhibit a moderate impact on *PAX6* functionality and are often associated with some atypical *PAX6*-associated phenotypes, such as mild forms of iris coloboma or isolated foveal hypoplasia, or more severe phenotypes of Peter’s anomaly and microphthalmia (Hanson et al., 1999; Azuma et al., 2003; Nallathambi et al., 2006; Jia et al., 2010; Thomas et al., 2014). However, no obvious genotype-phenotype correlations have been established to date (Gupta et al., 1998; Gronskov et al., 1999; Dubey et al., 2015; Sannan et al., 2017).

Aniridia, characterized by the incomplete development of the iris and fovea, is the most frequent *PAX6*-related condition, with a worldwide incidence of 1:50,000–100,000 births (Hingorani et al., 2012). Patients exhibit photophobia, low visual acuity, and nystagmus. Phenotypic variability is commonly observed (Lee and Colby, 2013). Iris hypoplasia may manifest as a complete absence of the iris (or “aniridia”) but also may present with minor structural iris defects, leading to atypical iris coloboma, iris holes, or stromal hypoplasia (Skeens et al., 2011), symptoms which can be only recognizable on slit-lamp examination. Additionally, a wide range of abnormalities in the cornea, anterior chamber, lens, and optic nerve have been reported conferring a higher risk in patients for secondary glaucoma, cataracts, and aniridia-associated keratopathy (AAK), which further worsen the visual outcome (Lee and Colby, 2013). Inter-familial and intra-familial variability on disease onset and severity of these secondary symptoms manifesting as congenital, childhood, or even adult forms are also observed (Hingorani et al., 2012; Vasilyeva et al., 2017). However, the molecular mechanisms underlying the variable expressivity of the *PAX6* mutations have yet to be elucidated. Identifying and understanding the genetic mechanisms that affect the severity of the disease is essential to provide a more accurate diagnosis and better clinical management of *PAX6*-associated disorders.

Somatic or gonosomal mosaicism might explain part of the phenotypic variability and/or reduced penetrance in aniridia, similarly to that reported in other dominant ocular disorders (Faivre et al., 2006; Beryozkin et al., 2016). Post-zygotic variants (PZV), arising as errors in DNA replication at early embryonic stages, have recently been revealed as a novel source of *de novo* and somatic mosaic variants thanks to the advent of more sensitive genotyping technologies (Acuna-Hidalgo et al., 2015, 2016). The developmental stage at which PZVs arise has a major influence on their frequency and distribution in affected tissues and thus on phenotypic expressivity and the recurrence risk in offspring (Acuna-Hidalgo et al., 2016). Interestingly, recent studies have showed that PZVs could account for up to 10% of rare neurodevelopmental disorders such as intellectual disability, epilepsy, and autism (Acuna-Hidalgo et al., 2015; Stosser et al., 2017; Myers et al., 2018); therefore, mosaicism may be underestimated in sporadic cases of other developmental diseases.

In aniridia, up to two-thirds of patients are sporadic (Netland et al., 2011; Lee and Colby, 2013) and thought to carry *de novo* mutations that are generally not detected in parental blood samples. Nevertheless, germline or even low-level somatic mosaicism in one of the parents cannot be ruled out, as multiple tissues are not usually tested during genetic screening. Germline mosaicism has long been postulated in *PAX6*-related disorders to explain the disease co-occurrence in several affected siblings with no family history of eye disorders (Reed and Falls, 1955; Gronskov et al., 1999; Deml et al., 2016; Riera et al., 2017). However, to our knowledge, *PAX6* mosaicism has never been reliably confirmed in a suspected family, mainly due to technical limitations in genetic testing and/or constraints on the availability of germinal or somatic DNA samples other than blood. To date, only a small number of exceptional cases of somatic mosaicism for 11p13 microdeletions have been reported in aniridia (Robinson et al., 2008; Erez et al., 2010; Huynh et al., 2017).

Here, we identify parental mosaicism in three families with *PAX6*-related ODAs in which gonosomal PZVs have been accurately assessed through quantitative droplet-digital PCR (ddPCR). Detection of mosaicism was very relevant to these families in terms of genetic counseling, which allowed for more accurate determination of the risk of recurrence in future offspring. Remarkably, our findings also highlight the inter-generational variable expressivity in these families, which seems to be clearly explained by the presence of parental mosaic *PAX6* mutations as well as by the further transmission of these mutations to the offspring. Thus, our work confirms that mosaicism is an underestimated cause of phenotypic variability and disease recurrence in aniridia and other *PAX6-*related ODAs.

## Materials and Methods

### Patients

We studied a cohort of 247 unrelated Spanish families with ODAs consisting of 78 with congenital aniridia, 33 with anterior segment dysgenesis and 136 with other *PAX6*-related phenotypes, i.e., ocular coloboma, microphthalmia, isolated foveal or optic nerve hypoplasia. Patients and affected and healthy relatives were recruited from two public hospitals from Madrid (Spain), Fundación Jiménez Díaz University Hospital and University Hospital Ramón y Cajal. Genomic DNA was obtained from peripheral blood, saliva, urine, and sperm using standard procedures.

This study was designed in compliance with the tenets of the Helsinki Declaration, and patient enrollment was approved by the ethics committees of both institutions. All participants or their legal guardians provided written informed consent prior to their participation in this study.

### Molecular Screening

Probands were screened according to a previously reported genetic algorithm for molecular diagnosis of *PAX6* defects (Blanco-Kelly et al., 2013). Pathogenic *PAX6* variants were screened by Sanger or next-generation sequencing (NGS). 11p13 microdeletions were studied by MLPA and/or custom CGH-arrays, as previously reported (Blanco-Kelly et al., 2017).

The proband of Family 1 was screened using a customized 151-gene panel (unpublished data). Both probands of Families 2 and 3 were studied by means of a custom 260-gene panel, as previously described (Ceroni et al., 2018). Briefly, library capture of all coding and non-coding exons and 20 bp of intronic boundaries was performed using HaloPlex or SureSelect QXT technologies (Agilent Technologies, Santa Clara, CA, United States). Massive sequencing was carried out using Illumina MiSeq or NextSeq 500 platforms running on paired-end mode at a minimum of 450X.

Bioinformatic analysis was performed using standard procedures and custom in-house pipelines for mapping, variant calling, and annotation. Pathogenicity prediction of missense variants was performed using CADD^1^, M-CAP^2^, and Alamut software (Interactive Biosoftware, France), which includes SIFT, Polyphen, MutationTaster, and Align GVGD. Population frequencies of the detected variants were assessed using gnomAD^3^ and CIBERER Spanish Variant Server^4^. Variants were also searched in the literature and in the *PAX6* database^5^. All variants detected by NGS were validated by Sanger sequencing using specifically designed primers (Available On Request). The mutation nomenclature was referred to the canonical RefSeq *PAX6* isoform NM_000280.4.

### SNaPshot Assays

Amplified *PAX6* exons 5, 6, and 10 were analyzed for the presence of c.120C>A, c.178T>C, and c.771G>A variants using the ABI Prism SNaPshot Multiplex kit (Themo Fisher). Reactions were performed in a 20 μL mix containing 3 μL purified PCR amplicons, 3 μL SNaPshot Ready Multiplex Ready Reaction Mix, and 0.4 μM of specific primers. Single base extension and further post-extension SAP treatment were performed following the manufacturer’s protocol. Labeled products were separated on an ABI3130xl Genetic Analyzer and analyzed with GeneMapper v4 software.

### ddPCR

Custom or commercial TaqMan SNP Genotyping Assays (Thermo Fisher) were used for genotyping the *PAX6* variants c.120C>A, c.178T>C, and c.771G>A (rs121907929; commercial Assay ID: C_152371166_10). DNA samples were evaluated by ddPCR using the Droplet Digital PCR QX200 System (Bio-Rad Laboratories, Hercules, United States) in a 20 μL PCR mix containing 10 μL 2x ddPCR Supermix for probes (Bio–Rad), 900 nM target-specific PCR primers, 250 nM FAM-labeled (mutant–allele) probe, and 250 nM VIC-labeled (wild–type allele) probe. Droplet emulsion was thermally cycled on a C1000 Touch Thermal Cycler (Biorad) in the following conditions: denaturing at 95°C for 10 min, 40 cycles of PCR at 94°C for 30 s, and a single step of annealing/extension at 56.4°C for 1 min, and a final step to deactivate enzyme at 98°C for 10 min. Each sample was run in quadruplicate. Data were analyzed using Quantasoft v1.7 software (Bio-Rad) using Rare Event Detection settings.

## Results

### Detection of Parental Mosaicism in PAX6

We investigated the presence of parental mosaicism associated with disease-causing *PAX6* variants in 3 families from a large cohort of Spanish patients with aniridia (*n* = 78) and other ODAs (*n* = 169). First, heterozygous *PAX6* variants were identified in probands of these 3 families through targeted NGS using custom approaches for *PAX6* screening. We found the previously reported nonsense variants c.120C>A;p.(Cys40^∗^) and c.771G>A;p.(Trp257^∗^) (Vincent et al., 2003; Graziano et al., 2007) in 2 probands with aniridia, and a novel likely pathogenic missense variant, c.178T>C;p.(Tyr60His), in a proband with non-syndromic microphthalmia. This last variant in the paired domain of PAX6 affected a highly conserved tyrosine residue (GERP ^++^ score 5.4 and PhyloP score 9.3), was predicted to be pathogenic by all *in silico* tools, had not been publicly reported in population and mutation databases, and segregated dominantly in two consecutive generations of affected individuals.

**FIGURE 1:**
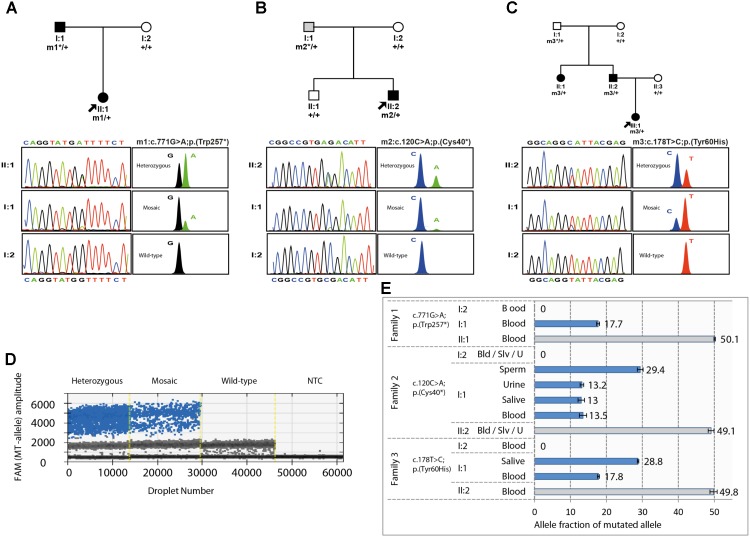
Pedigrees, familial segregation, and mosaicism analysis of the three families carrying the PAX6 variants. **(A–C)** Family pedigree and segregation analysis of the heterozygous and mosaic *PAX6* variants, c.771G>A (p.Trp257^∗^) from family 1 **(A)**; c.120C>A (p.Cys40^∗^) from family 2 **(B)**; and c.178T>C (p.Tyr60His) from family 3 **(C)** are shown. *PAX6* variants were numbered according to RefSeq transcript NM_000280.4, using for nucleotide numbering + 1 for the translation initiation codon. Individuals with congenital aniridia or microphthalmia are indicated with black symbols, and the mosaic individual with isolated iris coloboma is indicated in gray. Probands are indicated by arrows. Sanger and SNaPshot electropherograms are shown for wild-type, heterozygous, and mosaic individuals. m1, m2 and m3 represent mutated alleles, +, wild-type alleles and ^∗^ indicates mosaic alleles. **(D,E)**. Absolute quantification of the allele abundance by Digital Droplet PCR (ddPCR) assays using Taqman SNP Genotyping assays. **(D)** Example of the 1-D fluorescence amplitude plot of droplets for mutant allele detection in the FAM channel in Family 2 for the heterozygous carrier (II:2), the mosaic father (I:1), a wild-type homozygous carrier (I:2), and no template control (NTC). FAM-positive droplets (blue), containing the mutant allele, exhibit increased fluorescence compared to negative droplets (gray). **(E)** Allele fraction of mutated allele was calculated for the FAM-positive droplets *versus* VIC-positive droplets (wild-type alleles). Depending on tissue availability, somatic cells from blood (Bld), saliva (Slv), sperm and urine (U) samples were tested in the suspected mosaic individuals (I:1) from each family and compared to their respective probands and healthy individuals as fully heterozygous and wild-type controls. In Family 2, sperm cells from the mosaic father were tested to calculate the recurrence risk.

The occurrence of germinal or gonosomal mosaicism was suspected in 2 of the 3 families as the most plausible factor to explain either the unusual recurrence of *PAX6*-related microphthalmia in the offspring of a healthy couple (Family 3, **Figure 1C**), or the remarkable inter-generational phenotypic variability between a proband and her father (Family 2, **Figure 1B**), respectively. In this sense, Sanger sequencing revealed some traces of the mutant alleles (c.120C>A or c.178T>C) in paternal blood samples, for which the peak heights were substantially lower than those of their heterozygous children. Surprisingly, a third case of potential mosaicism was suspected in the affected father of Family 1. This individual displayed a similar biased sequencing pattern of the mutant allele c.771G>A (**Figure 1A**). Moreover, unequal amplification of the mutant and wild-type alleles was evidenced in all the suspected mosaic individuals when SNaPshot was used (**Figure 1**). Haplotyping analysis confirmed paternity in the three families (data not shown).

Mosaicism was finally evaluated by ddPCR using allele-specific Taqman genotyping, which enabled us to obtain a more accurate assessment of the allele imbalance by quantifying the mutant allele fraction (AF) (**Figure 1D**). We confirmed the presence of somatic mosaic *PAX6* alleles in these 3 individuals who showed variable AFs of 13–29% depending on the tested tissue (**Figure 1E**). As somatic mosaic variants were vertically transmitted to affected offspring in these families, we assumed that mutant alleles should also be present in the mosaic individuals in a relevant fraction of gonadal tissue. In this sense, we were able to confirm gonosomal mosaicism in the father of Family 2, detecting the mutant allele in gamete-forming cells at a ratio of 29%, as well as in several somatic tissues, such as blood, saliva, and urine cells, with a lower AF of 13%. As a result, we accurately established that the transmission risk of the variant p.(Cys40^∗^) in this family was about one-third. Unfortunately, sperm samples were unavailable in the other two mosaic fathers.

Overall, we have confirmed three cases of mosaicism in a cohort of 247 families with ODAs, representing a rate of 1.2%. Considering only aniridia, the mosaic rate is 3.8% with 2 families in a cohort of 78 families.

### Phenotypic Variability Associated With Mosaic PAX6 Mutations

**Table 1** summarizes all the currently available clinical data from mosaic fathers and their respective affected relatives. Fully heterozygous carriers of the nonsense *PAX6* variants presented classical symptoms of congenital aniridia, including marked iris hypoplasia, photophobia, nystagmus, low visual acuity, as well as congenital or early-onset cataracts. Additional progressive ocular features such as glaucoma and AAK underlying limbal insufficiency were later developed. The proband of family 1, a 29-year-old female carrying the variant p.Trp257^∗^, also suffered from mild growth retardation in childhood. However, she showed no signs of ataxia or intellectual disability, both systemic features previously reported in a patient with this same mutation (Graziano et al., 2007). Surprisingly, her affected father (I:1) also suffered from congenital aniridia and cataracts, despite carrying the mutant allele in only 18% of his blood cells. Unlike the daughter, he presented an apparently milder disease outcome with no signs of AAK at 50 years of age. By contrast, the mosaic carrier of the variant p.Cys40^∗^ (Family 2, I:1), with a slightly lower grade of mosaicism (13%) in several somatic tissues, showed neither congenital aniridia nor any apparent serious visual impairment. He was only diagnosed with iris coloboma and cataracts in both eyes in the fourth decade of life, after the birth of his son, who had aniridia.

**Table 1:** Genetic and clinical features of the families carrying postzygotic *PAX6* variants.

Family ID	Causing variant	Patient ID	Age	Genotype (mosaic AF)	Iris anomaly	Microphthalmia	Nystagmus	Congenital or early-onset cataracts	Glaucoma	Keratopathy	Other features
1	c.771G>A p.(Trp257^∗^)	I:1 (father)	50	Mosaic (18%, blood)	Complete aniridia (B)		+ (B)	+ (B)	+ (B)		Cataract surgery
		II:1	29	HET	Complete aniridia (B)		+ (B)	+(B)	+ (B)	Limbal insufficiency (B)	Growth retardation: cataract surgery; amaurosis (RE)
2	c.120C>A p.(Cys40^∗^)	I:1 (father)	59	Mosaic (13%, blood, saliva, and urine; 29%, sperm)	Coloboma (B)			+ (B)			
		II:2	25	HET	Complete aniridia (B)		+ (B)	+ (B)	+ (B)	Corneal opacity with limbal insufficiency (B)	Surgical interventions for limbal transplantation and implantation of Ahmed glaucoma valve (RE); BCVA: FC/0.05.
3	c.178T>C p.(Tyr60His)	I:1 (father)	80	Mosaic (18%, blood; 29%, saliva)							Asymptomatic; no ocular features
		II:1	43	HET		+ (B)	+(B)	+ (B)			High hyperopia; secondary corectopia after iridectomy.
		II:2	38	HET		+ (B)	+ (B)	+ (B)			High hyperopia; secondary corectopia after iridectomy.
		III:1	10	HET		+ (B)	+ (B)	+ (B)			High hyperopia; secondary corectopia after iridectomy.


*Nucleotide numbering reflects cDNA in the reference sequence NM_000280.4 for *PAX6*. AF, allele fraction; B, bilateral; BCVA, best corrected visual acuity; FC, finger counting; HET, heterozygous; ID, identification; and RE, right eye.*

The three heterozygous carriers of the missense p.(Tyr60His) variant presented a similar ocular phenotype of bilateral microphthalmia, congenital cataracts, nystagmus, and high hyperopia, without showing any systemic signs. Mosaicism was identified in the asymptomatic father (I:1, Family 3) of two affected siblings. Quantitative data from blood and buccal cells showed a relatively high mutant AF (18 and 29%, respectively). However, he has never complained of visual impairment, and his eyes were found to be normal in a thorough ophthalmic examination carried out in the 8th decade of life.

## Discussion

Phenotypic variability is a well-established phenomenon for *PAX6* defects (Hanson et al., 1999; Hingorani et al., 2012) that could be hypothetically attributable to genetic or epigenetic modifiers, pleiotropy, mosaicism, and/or gene dosage (Schedl et al., 1996; Hanson et al., 1999; Vincent et al., 2003; Chou et al., 2015; Cvekl and Callaerts, 2017; Yasue et al., 2017). Here, we present 3 families from our Spanish cohort of ODAs manifesting distinctly inter-generational differences in disease severity and/or ocular phenotype. We confirmed that the variable expressivity observed in all of these families resulted from parental mosaic *PAX6* mutations. Both families showed somatic mosaic alleles with a relatively high AF (> 10%) in healthy or mildly affected individuals and their vertical transmission to affected offspring, which suggests the existence of gonosomal PZVs. In one of them, gonosomal mosaicism was able to be assessed thanks to the availability of both somatic and gonadal samples, resulting in better estimation of the transmission risk of the mutation to future offspring. The estimated genetic risk of about one-third found in this family is quite higher than the risk level expected in a sporadic case carrying a *de novo* variant, which is usually considered to be negligible (Acuna-Hidalgo et al., 2016). In the other two families, the exact risk could not be assessed due to the unavailability of sperm samples. However, both fathers likely carried the pathogenic *PAX6* variant in a proportion of their germ line. Remarkably, the identification of mosaicism in an affected individual with aniridia, as we report here, involves modifying the initial genetic counseling, since the likelihood of recurrence is obviously lower than in heterozygous carriers. In view of our findings, parental mosaicism should be considered in sporadic cases with *PAX6*-associated diseases, even if parents do not show any ocular abnormalities.

In the two mosaic individuals carrying nonsense *PAX6* mutations, similar levels of somatic mosaicism led to variable clinical outcomes ranging from congenital aniridia to considerably milder manifestations. Interestingly, we are the first to confirm low-level mosaicism in an affected individual clinically diagnosed with congenital aniridia but who manifested some apparently milder secondary ocular features compared to his fully heterozygous daughter. However, the mosaic father of a second family only showed minor iris defects that went unnoticed until adulthood. Therefore, mosaicism levels in blood are not a reliable indicator of the range and severity of ocular features. The variable phenotypic expression observed in these mosaic individuals could be influenced by different factors, including specific mutational effects, gene dosage, and lineage and timing-dependent *PAX6* functionality, not only during ocular development but also in adult tissues (Shaham et al., 2012; Gregory-Evans et al., 2014; Cvekl and Callaerts, 2017). In this sense, previous studies with transgenic and, more recently, genetically edited animal models confirmed that the patterning and differentiation of different ocular structures and cell lineages require different thresholds of *PAX6* activity throughout eye embryogenesis (Davis-Silberman et al., 2005; Yasue et al., 2017). Specifically, somatic *PAX6* mosaicism in CRISPR-edited mouse embryos caused varying degrees of ODAs, as recently published (Yasue et al., 2017). Therefore, the identification of mosaic individuals for *PAX6* mutations both in humans and animal models manifesting variable expressivity strongly reinforces the idea that *PAX6* dosage plays a specific role in both eye development and phenotypic modulation. According to our findings and those reported using CRISPR models, somatic *PAX6* mosaicism might explain some of the approximately 10% of patients with aniridia who apparently lack *PAX6* mutations (Ansari et al., 2016; Bobilev et al., 2016; Vasilyeva et al., 2017) or even mild cases of ODAs (Hanson et al., 1999). The widespread use of more sensitive techniques, such as targeted deep sequencing and/or ddPCR, will allow for the identification of additional cases with somatic or gonosomal PZVs arising at early stages (Acuna-Hidalgo et al., 2016). However, due to limitations on tissue sampling, it will remain challenging to detect PZVs with low AF that could appear later in embryonic development or those restricted to ocular tissues.

It has long been postulated that germline mosaicism in healthy parents could be the most likely explanation for the exceptional disease recurrence in affected siblings with no family history of aniridia (Reed and Falls, 1955; Gronskov et al., 1999) or *PAX6*-related microphthalmia (Deml et al., 2016; Riera et al., 2017). To date, this possibility has not been fully confirmed due to the limited availability of somatic and gonadal tissues for genetic testing. Here, we report an apparent autosomal dominant 3-generation family with microphthalmia in which we first assumed a reduced penetrance due to both disease recurrence in the first affected siblings and healthy parents. However, NGS analyses only revealed a novel likely pathogenic missense variant in *PAX6*, a highly penetrant gene. Similar missense mutations in the paired-domain of *PAX6* have been occasionally reported to cause microphthalmia (Dansault et al., 2007; Chassaing et al., 2013; Riera et al., 2017; Patel et al., 2018); thus, this variant seemed to be the cause of the phenotype in the family. To delve deeper into the causes of this unusual disease recurrence, we exhaustively investigated the possibility of parental germline mosaicism in the unaffected first generation. In the asymptomatic father of this family, an unexpectedly high mutant AF was detected in several somatic tissues from ectoderm and endoderm lineages indicating that the mosaic PZV had to emerge early in the first embryonic cell divisions. Additionally, the lack of ocular signs in this mosaic individual suggests that the PAX6 activity had to reach an adequate threshold through optic cup morphogenesis. We could not discern whether this fact was related to a gene dosage effect underlying a lower mutant ratio in embryonic eye or if this variant is hypomorphic, similar to that described for other missense *PAX6* variants (Tang et al., 1997; Vincent et al., 2003). Despite the rarity of *PAX6*-associated microphthalmia, with fewer than 10 variants described to date (Deml et al., 2016), it is worth noting that disease recurrence has putatively been associated with mosaicism in a healthy progenitor in two additional families (Deml et al., 2016; Riera et al., 2017). Thus, the possibility of *PAX6* mosaicism should also be considered when explaining occurrence in two or more affected siblings with microphthalmia in the absence of family history.

Recent evidence has suggested that transmission of parental mosaicism could explain up to 10% of apparently *de novo* mutations in some pathologies, and thus mosaicism rates might be higher than previously expected (Acuna-Hidalgo et al., 2016; Stosser et al., 2017). In our cohort of patients with aniridia, mosaic PZVs were identified in at least 3.8% of families, though this rate is likely underestimated. We cannot rule out that some of our patients without *PAX6* mutations or some parents of *de novo* cases might present a low-grade of mosaicism. Therefore, larger systematic studies should be carried out using high-sensitivity ultradeep sequencing, single cell analysis or ddPCR in several somatic tissues to obtain a better estimation of the actual mosaicism rate as well as to elucidate the actual relevance of PZVs in the pathogenesis of *PAX6*-associated diseases.

In brief, our work confirms the long-held hypothesis of germinal mosaicism in aniridia to explain disease recurrence in the offspring of healthy parents. Additionally, we demonstrate for the first time that mosaic *PAX6* variants cause phenotypic variability from congenital aniridia, mild forms of isolated iris coloboma, and even asymptomatic individuals. Consequently, our findings have important clinical implications for genetic counseling and ophthalmic management in congenital aniridia and related developmental eye disorders. Finally, somatic and/or gonosomal mosaicism should be taken into consideration as genetic factors to explain not only extraordinary cases of co-occurrence in siblings but also intra-familial variable expressivity, as well as some of apparently *de novo* cases and even uncharacterized *PAX6*-negative cases in aniridia.

## Author Contributions

MC and MM contributed the conception and design of the study. MT, MM, PR, and CV performed genetic analyses. MT, MM, PR, M-AM-P, and MV participated in the analysis and interpretation of genetic data for the work. MG, FB-K, DR-Z, GR, FM-N, ST-S, BG, and CA participated in the recruitment, clinical evaluation, and interpretation of clinical data. MC wrote the first draft of the manuscript. MT, MM, and MV wrote and/or prepared sections of the manuscript. All authors contributed to manuscript revision, reading and approving the submitted version.

## Conflict of Interest Statement

The authors declare that the research was conducted in the absence of any commercial or financial relationships that could be construed as a potential conflict of interest.

## Footnotes

1 http://cadd.gs.washington.edu/

2 http://bejerano.stanford.edu/mcap/

3 http://gnomad.broadinstitute.org/

4 http://csvs.babelomics.org/

5 http://lsdb.hgu.mrc.ac.uk/home.php?select_db=PAX6
